# Plasmid-mediated Quinolone Resistance among Non-Typhi*Salmonella enterica* Isolates, USA

**DOI:** 10.3201/eid1611.100464

**Published:** 2010-11

**Authors:** Maria Sjölund-Karlsson, Rebecca Howie, Regan Rickert, Amy Krueger, Thu-Thuy Tran, Shaohua Zhao, Takiyah Ball, Jovita Haro, Gary Pecic, Kevin Joyce, Paula J. Fedorka-Cray, Jean M. Whichard, Patrick F. McDermott

**Affiliations:** Author affiliations: Centers for Disease Control and Prevention, Atlanta, Georgia, USA (M. Sjölund-Karlsson, K. Joyce, J.M. Whichard); IHRC, Inc., Atlanta (R. Howie, G. Pecic);; Atlanta Research and Education Foundation, Decatur, Georgia, USA (R. Rickert, A. Krueger);; Food and Drug Administration, Laurel, Maryland, USA (T.-T. Tran, S. Zhao, P.F. McDermott);; US Department of Agriculture, Athens, Georgia (T. Ball, J. Haro, P.J. Fedorka-Cray)

**Keywords:** *Salmonella enterica*, bacteria, antimicrobial drug resistance, fluoroquinolones, quinolone resistance, United States, dispatch

## Abstract

We determined the prevalence of plasmid-mediated quinolone resistance mechanisms among non-Typhi *Salmonella* spp. isolated from humans, food animals, and retail meat in the United States in 2007. Six isolates collected from humans harbored *aac(6′)Ib-cr* or a *qnr* gene. Most prevalent was *qnrS1*. No animal or retail meat isolates harbored a plasmid-mediated mechanism.

Severe *Salmonella enterica* infections are commonly treated with fluoroquinolones (e.g., ciprofloxacin) (*1*). In the United States, the antimicrobial drug susceptibility of *Salmonella* spp. isolated from humans, food animals, and retail meats is systematically monitored by the National Antimicrobial Resistance Monitoring System (NARMS). This program is a collaborative effort of the Centers for Disease Control and Prevention (CDC), the Food and Drug Administration Center for Veterinary Medicine (FDA-CVM) and the US Department of Agriculture (USDA). Antimicrobial susceptibility to fluoroquinolones among *Salmonella* spp. has been monitored since the program’s inception in 1996.

Although fluoroquinolone resistance in *Enterobacteriaceae* is predominantly due to topoisomerase mutations, 3 plasmid-mediated mechanisms have been described that confer decreased susceptibility to ciprofloxacin: quinolone resistance proteins (Qnr), Aac(6′)-Ib-cr, and QepA efflux (*2*). The Qnr proteins protect the DNA-gyrase from quinolones, Aac(6′)-Ib-cr modifies quinolones with a piperazinyl group, and QepA is involved in active efflux (*2*). Because patients have experienced treatment failure when infected with *Salmonella* isolates that displayed decreased susceptibility to fluoroquinolones, plasmid-mediated mechanisms are clinically relevant (*3*).

A survey of 12,253 NARMS non-Typhi *Salmonella* (NTS) isolates collected from humans from 1996 through 2003 identified 10 (0.08%) *qnr*-positive isolates (*4*). A second survey of NARMS NTS collected from humans during 2004–2006 showed an increase in the proportion of isolates harboring plasmid-mediated quinolone resistance mechanisms. Among 6,057 isolates, 17 *qnr-*positive isolates and 1 *aac(6*′*)-Ib-cr*-positive isolate were detected, representing 0.3% of the NTS collected during that time (*5*).

The increase in plasmid-mediated quinolone resistance among NTS isolated from humans in the United States prompted further studies to determine continued presence among NTS of human origin and possible reservoirs of these mechanisms. In this study, we investigated plasmid-mediated quinolone resistance mechanisms among NARMS NTS isolated from humans, food animals, and retail meat in the United States in 2007.

## The Study

In 2007, 54 NARMS-participating public health laboratories from all 50 states forwarded every 20th human isolate of NTS to CDC. Similarly, NTS isolated from retail meat (chicken breasts, ground turkey, ground beef, and pork chops) were submitted by 10 states that participated in CDC’s Foodborne Diseases Active Surveillance Network (FoodNet) for analysis at FDA-CVM. NTS from food animals were obtained from carcass rinsates (chicken), carcass swab specimens (turkey, cattle, and swine), and ground products (chicken, turkey, and beef). Animal samples were collected by the Food Safety Inspection Service of the USDA from federally inspected slaughter and processing plants throughout the United States and sent to USDA facilities in Athens, Georgia, for further analysis.

At each agency, MICs were determined by broth microdilution (Sensititer; Trek Diagnostics, Westlake, OH, USA). Human, animal, and retail meat isolates of NTS that displayed decreased susceptibility to ciprofloxacin (MIC >0.25 mg/L) were included in our study. For each isolate, genomic DNA was prepared by lysing the bacteria at 95°C and collecting the supernatant after centrifugation. PCRs with previously described primers were used to screen isolates for *qepA*, *aac(6*′*)-Ib-cr,* and *qnr* genes (*qnrA, B, C, D, S*) (*6*–*10*). Positive controls were included for *qepA* (*Escherichia coli* TOP10 pAT851), *qnrA* (*S. enterica* serotype Montevideo AM28704), *qnrB* (*S. enterica* serotype Berta AM04589), *qnrS* (*S. enterica* serotype Bovismorbificans AM12888) and *aac(6*′*)-Ib-cr* (*E. coli* 36564). For isolates with positive results in the screening, amplicons were confirmed by direct sequencing by using a 3730 DNA Analyzer (Applied Biosystems, Foster City, CA, USA).

Among 2,165 isolates of NTS collected from humans in 2007, 51 (2.4%) displayed decreased susceptibility to ciprofloxacin. Among 320 NTS obtained from retail meat, 5 (1.6%) showed decreased susceptibility to ciprofloxacin, and among the 1,915 isolates obtained from animal sources, 5 (0.3%) showed such susceptibility. Six (11.8%) of the 51 human isolates carried a plasmid-mediated mechanism that affected quinolones; 5 isolates harbored a *qnr* gene, and 1 isolate contained the *aac(6*′*)-Ib-cr* gene (Table). None of the isolates harbored the *qepA* gene. Sequencing of the 5 *qnr*-positive isolates showed 3 *qnrS* and 2 *qnrB* variants among 4 serotypes (Beaudesert, Corvallis, Enteritidis, and Typhimurium) (Table). The *aac(6’)-Ib-cr* gene was found in an isolate of serotype Thompson, and sequencing confirmed the 2 point mutations (Trp102Arg and Asp179Tyr) characteristic of the ciprofloxacin-modifying variant. The MIC of ciprofloxacin among the *qnr-*positive isolates ranged from 0.25 mg/L to 0.5 mg/L, whereas the *aac(6*′*)-Ib-cr*–positive isolate displayed an MIC of 0.5 mg/L. All isolates from humans were susceptible to nalidixic acid (MIC range 8–16 mg/L). None of the isolates obtained from retail meat or those isolated from animal sources harbored plasmid-mediated mechanisms affecting quinolones. However, all retail meat and animal isolates with decreased susceptibility to ciprofloxacin were resistant to nalidixic acid (MIC >32 mg/L), which suggests the presence of topoisomerase mutations.

**Table:** Characteristics of non-Typhi *Salmonella enterica* isolates harboring *qnr* or the *aac(6′)-Ib-cr* gene, collected through NARMS, 2007*

Isolate no.	*S. enterica* serovar	Submitting site	Resistance phenotype	Ciprofloxacin MIC, mg/L	*aac6′Ib/qnr* variant
AM31035	Thompson	NY	AMP, SUL	0.5	*aac(6′)-Ib*-cr
AM30827	Typhimurium	CA	STR, SUL, TET	0.5	*qnrS1*
AM31228	Corvallis	VA	ND	0.5	*qnrS1*
AM33914	Typhimurium	NC	ND	0.5	*qnrS1*
AM31434	Enteritidis	LX	SUL, SXT, TET	0.25	*qnrB2*
AM33097	Beaudesert	CA	ND	0.25	*qnrB19*

*NARMS, National Antimicrobial Resistance Monitoring System; NY, New York; AMP, ampicillin; SUL, sulfamethoxazole or sulfisoxazole; CA, California; STR, streptomycin; TET, tetracycline; VA, Virginia; ND, none detected; NC, North Carolina; LX, Los Angeles; SXT, trimethoprim/sulfamethoxazole.

The 6 patients (3 male and 3 female) who were infected with a Qnr-producing or Aac(6′)-Ib-cr–producing *Salmonella* isolate had a median age of 18 (range 3–84 years). Three patients were available for interview. They reported gastrointestinal symptoms and had sought medical care for their condition. Two of the patients had received antimicrobial drug treatment (ciprofloxacin and cefdinir, respectively); none of the patients developed an invasive infection. Two patients reported a history of international travel to Mexico and Thailand, respectively.

## Conclusions

Six (0.3%) NARMS NTS collected from humans in 2007 harbored a plasmid-mediated quinolone resistance mechanism, the same prevalence as in 2004–2006 (*5*). None of the isolates collected from animal and retail meat by the USDA and FDA in 2007 harbored these mechanisms. Among the human isolates, *qnr* genes predominated and *qnrS1* was most prevalent. This gene has previously been described among NARMS human NTS and was first detected in an isolate of serotype Bovismorbificans collected in 2000 (*4*). The gene was later reported in 11 isolates (serotypes Corvallis, Enteritidis, Montevideo, Saintpaul, and Typhimurium) collected by NARMS during 2004–2006 (*5*).

That *qnr* genes could only be detected among *Salmonella* isolates obtained from humans warrants further exploration. One factor that could influence the number of Qnr-producing *Salmonella* isolates among humans in the United States is the extent of travel-associated infections. Two patients in this study had a history of international travel before illness onset. Another factor that could lead to the development of Qnr-producing *Salmonella* isolates is the in vivo transfer of resistance from other *qnr*-bearing *Enterobacteriaceae.*

Our study does not suggest that food animals and meat in the United States are major sources of *Salmonella* isolates that harbor plasmid-mediated quinolone resistance mechanisms. However, animals and food have been described as reservoirs for these mechanisms elsewhere. A high prevalence of *Enterobacteriaceae* with *qnr* and *aac(6’)-Ib-cr* have been reported among companion and food animals in the People’s Republic of China and *qnr-*positive *Salmonella* isolates have been found in poultry in Europe (*11*,*12*). Thus, other food and meat sources, not investigated in the current study, may serve as reservoirs for these mechanisms.

Fluoroquinolone resistance among isolates of NTS has important public health implications because ciprofloxacin is commonly used to treat invasive infections of *Salmonella* spp. in adults. Although plasmid-mediated quinolone resistance mechanisms do not, by themselves, confer clinical resistance to ciprofloxacin, they may promote the selection of mutations that do (*13*). In addition, studies have shown that patients infected with isolates that display low-level fluoroquinolone resistance may respond poorly to treatment, prompting a reconsideration of MIC breakpoints in clinical medicine (*3*,*14*). To avoid further dissemination of plasmid-mediated quinolone resistance among *Salmonella* and other *Enterobacteriaceae* isolates in the United States, prudent use of antimicrobial agents in both human and veterinary medicine will be crucial. Continued surveillance for resistant bacteria among human, animal, and food sources remains critical.
